# Translational implications of bradyarrhythmia in hibernating brown bears

**DOI:** 10.14814/phy2.15550

**Published:** 2023-01-03

**Authors:** Lisa A. Gottlieb, Alina L. Evans, Boris Fuchs, Ole Fröbert, Anna Björkenheim

**Affiliations:** ^1^ Department of Cardiology Copenhagen University Hospital – Bispebjerg Copenhagen Denmark; ^2^ Department of Biomedical Sciences, Faculty of Health and Medical Sciences University of Copenhagen Copenhagen Denmark; ^3^ Department of Forestry and Wildlife Management Inland Norway University of Applied Sciences Koppang Norway; ^4^ Department of Cardiology, Faculty of Medicine and Health Örebro University Örebro Sweden; ^5^ Steno Diabetes Center Aarhus Aarhus University Hospital Aarhus Denmark; ^6^ Department of Clinical Medicine, Faculty of Health Aarhus University Aarhus Denmark; ^7^ Department of Clinical Pharmacology Aarhus University Hospital Aarhus Denmark

**Keywords:** bradyarrhythmia, brown bear, hibernation, sinus arrest, sinus node disease, sinus pause, syncope, translational model, *Ursus arctos*

## Abstract

The brown bear *Ursus arctos* undergoes exceptional physiological adaptions during annual hibernation that minimize energy consumption, including profound decrease in heart rate, cardiac output, and respiratory rate. These changes are completely reversible after the bears reenter into the active state in spring. In this case report, we show episodes of sinus arrest in a hibernating Scandinavian brown bear and in humans, recorded by implantable loop recorders and discuss the possible underlying mechanisms. Lessons learned from cardiac adaptations in hibernating bears might prove useful in the treatment of patients with sinus node dysfunction.

## Graphical abstract

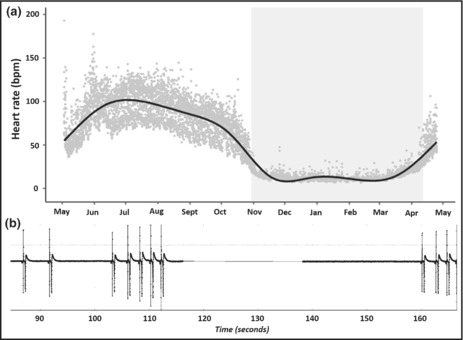

## 1 INTRODUCTION

The brown bear *Ursus arctos* has a resting heart rate similar to that of humans in its active state but during annual hibernation undergoes exceptional physiological adaptions that minimize energy consumption, including profound decrease in heart rate, cardiac output, and respiratory rate (Fröbert et al., 2020; Jørgensen et al., 2020; Laske et al., 2011). In hibernating bears, heart rate is approximately one third of that in active state, with frequent sinus arrests and extreme respiratory sinus arrhythmias (Gandolf et al., 2010; Laske et al., 2018; Tøien et al., 2011). Sinus arrest is a manifestation of sinus node dysfunction (SND) and its occurrence is the most common indication for permanent pacemaker therapy in symptomatic humans.

In this case report, we present SND in a free‐ranging Scandinavian brown bear and in two humans, documented by implantable loop recorders (ILR). Comparison of heart rhythm in brown bears and humans and an understanding of the mechanisms underlying adaptations in heart rhythm in hibernating brown bears can have translational implications for the treatment of SND in humans.

## 2 CASE REPORTS

### 2.1 Brown bear

A free‐ranging male brown bear (28 kg) born in 2018 was captured in May 2019 in Dalarna, Sweden. Under general anesthesia, the bear was marked with a global positioning system collar and a very high frequency transmitter, and implanted with a subcutaneous ILR (Reveal XT®, Medtronic Inc.) in the parasternal region as part of the Scandinavian Brown Bear Research Project (https://bearproject.info/). The research was approved by the local ethics authorities (Dnr 5.8 18–03376/2020) and is in compliance with the EU Directive 2010/63/EU for animal experiments. It has been described in detail (Jørgensen et al., 2020). A customized program (BearWare, Medtronic Inc.) was deployed in the ILR device and heart rate was recorded at two‐minute intervals (Laske et al., 2018).

**FIGURE 1:**
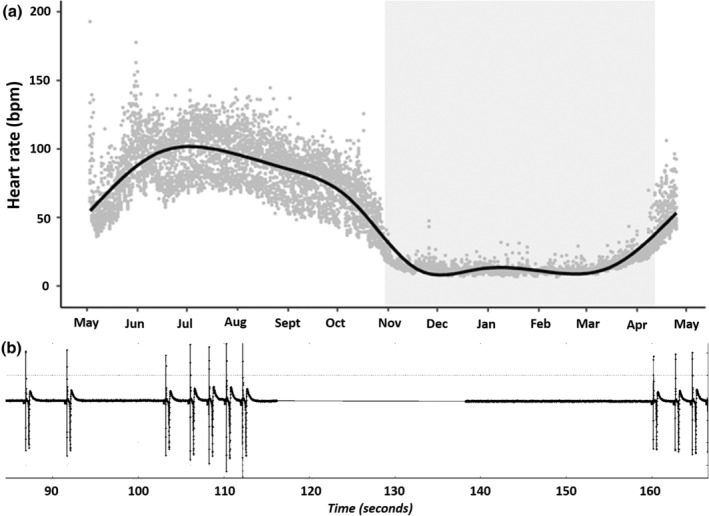
Heart rate and sinus arrest in a sub‐adult brown bear. (a) Heart rate in beats per minute (bpm), measured in a free‐ranging brown bear (*Ursus arctos*) in Sweden using an ILR implanted in May 2019. Data are displayed as hourly means (gray circles) and an estimated time dependent mean (solid line). The hibernation period based on inactivity is indicated as a shaded area. (b) A sinus arrest of 48 s during hibernation.

In its active state, the bear exhibited a heart rate of 24–255 bpm (mean 84 ± 25). During the ensuing hibernation period, heart rate varied from 5 to 136 bpm (mean 14 ± 8) (Figure 1a). Seventy‐three episodes of sinus arrest occurred during hibernation, the longest of which (48 s) occurred in mid‐January at 10.31 h (Figure 1b). The bear was recaptured in April 2020 and then weighed 46 kg. A hunter legally shot the bear in August 2020.

### 2.2 Human A

**FIGURE 2:**
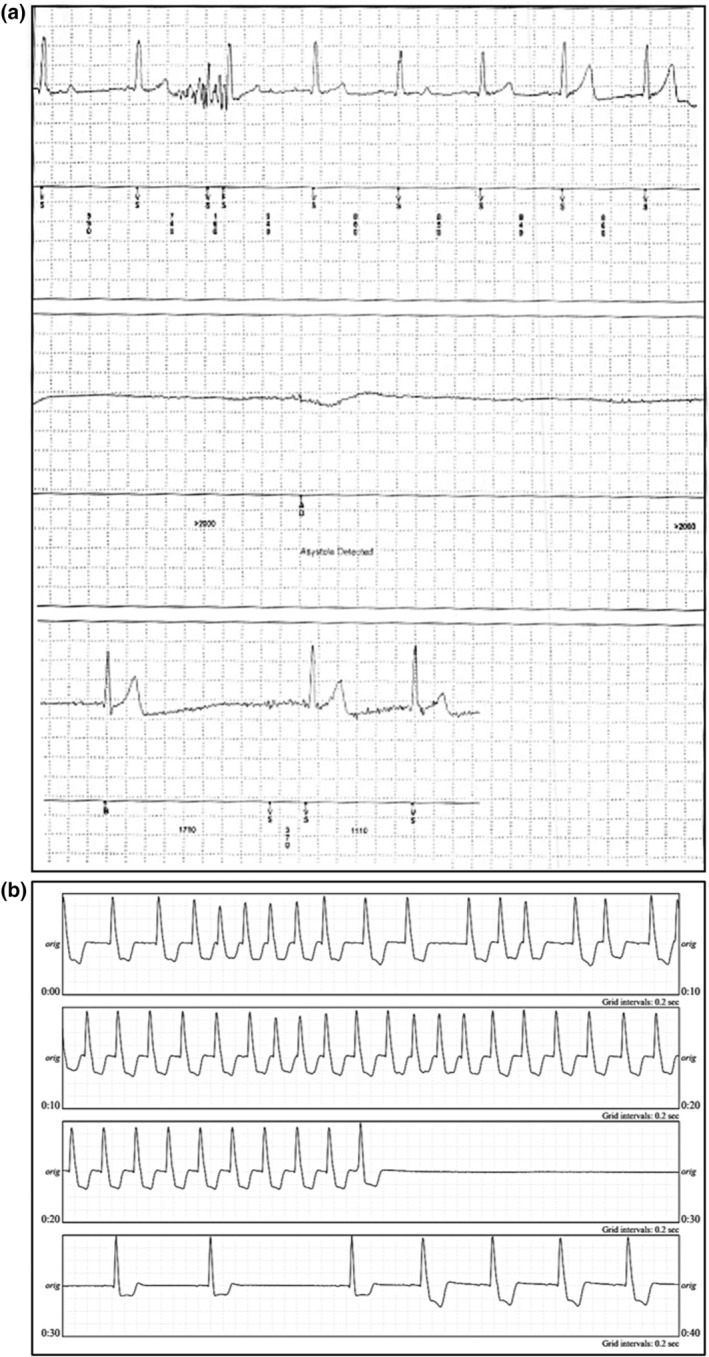
Sinus arrest in patients with continuous ECG monitoring following syncope. (a) Sinus arrest of 8.4 s in a now 61‐year‐old man with previous syncope. (b) Atrial fibrillation followed by a sinus arrest of 6.0 s in a 66‐year‐old woman.

A 60‐year‐old male truck driver exhibiting obesity, hypertension, and type 2 diabetes mellitus presented to the emergency department in Örebro University Hospital in 2012 following syncope while sitting. Results of physical examination, 12‐lead ECG, and routine blood samples were within normal limits. In‐hospital telemetry, transthoracic echocardiogram, exercise testing, coronary angiogram, careful carotid sinus massage, and computed tomography scan of the brain showed no pathology. Tilt testing showed a drop in systolic blood pressure from 135 to 62 mmHg. The patient experienced dizziness during the test but no loss of consciousness. The heart rate did not change; indicating autonomic nervous dysfunction. An invasive electrophysiological study showed no pathology, and an ILR (Reveal XT®, Medtronic Inc.) was implanted. The patient had a mean heart rate of 75 bpm (range 45–100 bpm). Because of his high‐risk occupation, the diagnostic work‐up was unusually extensive for a single unexplained syncope episode. One year later the patient experienced a symptomatic sinus arrest of 8.4 s during daytime, captured by the ILR (Figure 2a). A permanent pacemaker was subsequently implanted. The patient's condition has been monitored in the outpatient clinic with the most recent follow‐up in September 2022 and has not exhibited symptoms since the pacemaker implant.

### 2.3 Human B

A 66‐year‐old woman with paroxysmal atrial fibrillation for 11 years and treatment with betablockers and flecainid presented with syncope. In‐hospital telemetry showed no arrhythmias. The patient was implanted with an ILR (Reveal Linq, Medtronic) at the Department of Cardiology, Örebro University Hospital in June 2021. The following day the patient experienced another syncope event, and the ILR showed sinus arrest of 6 seconds at termination of atrial fibrillation (Figure 2b). The patient was implanted with a permanent pacemaker the following day and underwent atrial fibrillation ablation in May 2022.

## 3 DISCUSSION

We describe episodes of sinus bradycardia and extreme sinus arrest in a free‐ranging sub‐adult brown bear during hibernation and two cases of SND in patients. Continuous rhythm monitoring over an extensive period of time provides an opportunity to study spontaneous arrhythmias in brown bears during both active state and hibernation without potential bias introduced by captivity or anesthesia (Laske et al., 2011). In humans, ILRs are mainly used in patients with unexplained syncope, and documentation of bradyarrhythmia is sought before pacemaker implantation. The underlying mechanisms of SND in bears and humans may be similar, since mammals exhibit overall similarities in cardiac anatomy, conduction system, and physiology (Meijler & Meijler, 2011). However, in hibernating bears, bradyarrhythmias can be seen as a physiologically appropriate response to decreased metabolic demand, while in humans such processes are considered pathological when symptoms occur.

### 3.1 Heart rhythm and rate

In both humans and brown hears, sinus rhythm originates in the sinus node where cells are capable of pacemaker activity via funny current and calcium release (Quinn & Kohl, 2021). Impulse initiation, and hence the heart rate, depends on autonomic activation and local myocardial stretch (preload) (Quinn & Kohl, 2021). The normal resting heart rate in human adults ranges from 40 to 107 bpm and is generally higher during the day than at night (Rijnbeek et al., 2014). In bears, the heart rate during the active state resembles that of humans (mean 71 bpm) but, during hibernation, can drop to lower than 10 bpm and is without circadian variation (Jørgensen et al., 2020; Laske et al., 2011). In both brown bears and humans, heart rate seems to be independent of body mass (Gandolf et al., 2010).

### 3.2 Sinus arrhythmia, sinus bradycardia, and sinus node dysfunction

Sinus arrhythmia and sinus bradycardia are usually normal phenomena in humans. Sinus bradycardia occurs in healthy humans as an adaptive response, particularly in athletes with a pronounced increase in parasympathetic tone at rest or while sleeping, when heart rate may transiently drop to as low as 30 bpm (Kusumoto et al., 2019). Lack of sinus arrhythmia is common with increasing age and comorbidities due to reduced carotid distensibility and baroreflex sensitivity (Kaushal & Taylor, 2002).

In bears, extreme respiratory sinus arrhythmia is documented during hibernation when parasympathetic tone is high and may further reduce energy consumption, because heart rate increases only when oxygen levels rise during inspiration (Laske et al., 2018; Tøien et al., 2011). Respiration also influences venous return, periodically altering sinus nodal stretch and baroreceptor activity via blood pressure variation, ultimately modulating the heart rate (Quinn & Kohl, 2021).

Sinus bradycardia can also occur as a pathologic response as a manifestation of SND (Kusumoto et al., 2019). SND is caused either by the intermittent failure to generate automatic activation of the sinus nodal cells (sinus arrest) or by block of the impulse from the node to the atrium (sinoatrial block) (Kusumoto et al., 2019). As long as the physiological adaptation of increased stroke volume compensates for the decrease in heart rate, humans and other mammals with profound bradycardia remain asymptomatic. However, SND can result in fatigue and syncope when the physiological demands of blood supply are unmet (Kusumoto et al., 2019).

Mutations in genes coding for the pacemaker hyperpolarization‐activated cyclic‐nucleotide‐gated channel (HCN4) and sodium voltage‐gated channel alpha subunit 5A, reducing channel function, have been associated with SND in younger individuals (Milanesi et al., 2006; Wilde & Amin, 2018). Replacement of sinus node tissue by fibrous tissue often underlies SND in elderly humans (Choudhury et al., 2015). The structural remodeling can be triggered by common morbidities, including heart failure and diabetes mellitus (Choudhury et al., 2015), which probably played a role in patient A. Atrial arrhythmias can also result in adverse remodeling potentially leading to SND, as in patient B (Hocini et al., 2003). Noteworthy, in patients with SND, recovery of sinus node function has been demonstrated after cure of atrial fibrillation by ablation, indicating a potential for reverse remodeling of sinus node tissue (Hocini et al., 2003).

### 3.3 Translational model

Brown bears undergo several months of annual hibernation without eating or drinking as a survival strategy for conserving energy during winter (Fröbert et al., 2020). The underlying processes of physiological adaptation during hibernation are complex and involve metabolic rate depression despite only mild hypothermia (33–34°C), a switch from carbohydrate to lipid metabolism, and reduction in respiratory rate and oxygen consumption, despite retaining physiological control mechanisms (Fröbert et al., 2020). Specific cardiac adaptations in the hibernating state involve reduced cardiac output, bradycardia, respiratory sinus arrhythmia, and sinus arrest to reduce metabolic expenditure in heart tissue (Gandolf et al., 2010; Jørgensen et al., 2020; Laske et al., 2011, 2018; Tøien et al., 2011).

In humans, anorexia nervosa and hypothermia are states similar to hibernation. The exceptionally low caloric intake in anorexia nervosa is associated with heart rates as low as 25 bpm, sinus arrests, and increased heart rate variability that cease upon restoration of adequate diet (Kossaify, 2010; Mont et al., 2003). The cause of the bradyarrhythmia appears to be increased parasympathetic activity with unchanged sympathetic tone (Mont et al., 2003).

In humans, contrary to brown bears, mild hypothermia is associated with a marked increase in metabolic rate, increased respiratory rate, and tachycardia. Sinus bradycardia and ventricular arrhythmias are manifested when hypothermia progresses to moderate to severe (Mallet, 2002). Brown bears thus seem to adapt more rapidly to lower core temperatures than do humans. Studies of brown bears and other hibernating mammals show that alterations in heart rate and other physical processes during hibernation are fully reversible upon resumption of the active state (Jørgensen et al., 2020; Laske et al., 2011; Tøien et al., 2011). Bradyarrhythmia in hypothermia and anorexia nervosa in humans is also shown to be reversible (Kossaify, 2010; Mallet, 2002; Mont et al., 2003).

In both humans and bears, the autonomic nervous system plays an important role in the regulation of heart rhythm. The main adaptation mechanism during hibernation is probably high, centrally mediated, parasympathetic tone, but changes in sensitivity and downstream responses of autonomic activity are likely also involved. Low resting heart rates (<40 bmp) in endurance athletes are often explained by enhanced parasympathetic activity compared to non‐athletes (Guasch & Mont, 2017). Symptomatic syncope is rare in athletes because the metabolic demand is met. However, parasympathetic activity can also trigger atrial arrhythmias such as atrial fibrillation (Guasch & Mont, 2017). In summary, autonomic imbalance impacts SND.

In addition to parasympathetic enhancement, expression of certain genes in the sinus node, such as those coding for pacemaker and sodium channels and their regulators, is likely reduced during hibernation compared to the active state. Identification of such genes in bears could have implications for treatment of SND in humans. It can also be speculated that seasonal variations in collagen accumulation in the sinus node that attenuate sinus nodal‐atrial conduction occur in bears during hibernation. Such remodeling, and its reversion, could be gene‐modulated by altering the activity of matrix metalloproteinases and their inhibitors, known to influence collagen metabolism (Guasch & Mont, 2017).

Currently, treatment of SND in humans is directed at symptoms and involves implantation of a permanent pacemaker with risks of procedural complications and infections. In the future, gene therapy to treat SND in humans might be administered before the patient experiences symptoms, possibly using gene transfer vectors or CRISPR technology. Lessons learned from cardiac adaptations in hibernating bears might prove useful.

## 4 CONCLUSIONS

Study of mechanisms underlying arrhythmias and physiological adaptations during bear hibernation can yield insight and potentially provide a translational model that can be applied to humans. The main adaptation mechanism during hibernation is probably high centrally mediated parasympathetic tone, but rapid changes in expression of genes coding for pacemaker and sodium channels in the sinus node are likely also involved. This suggests possibilities for gene therapy strategies to treat SND.

## FUNDING INFORMATION

This research did not receive any specific grant from funding agencies in the public, commercial, or not‐for‐profit sectors.

## CONFLICT OF INTEREST

The authors have no conflicts to disclose.

## ETHICS APPROVAL

All procedures performed in this study were in accordance with the ethics standards of the 1964 Declaration of Helsinki and its later amendments or comparable ethical standards.

## PATIENT CONSENT STATEMENT

Written informed consent was obtained from the patients for the publication of this study.
